# Proliferation of Highly Cytotoxic Human Natural Killer Cells by OX40L Armed NK-92 With Secretory Neoleukin-2/15 for Cancer Immunotherapy

**DOI:** 10.3389/fonc.2021.632540

**Published:** 2021-04-15

**Authors:** Meng Guo, Chen Sun, Yuping Qian, Liye Zhu, Na Ta, Guangjian Wang, Jianming Zheng, Fengfu Guo, Yanfang Liu

**Affiliations:** ^1^ National Key Laboratory of Medical Immunology & Institute of Immunology, Navy Medical University, Shanghai, China; ^2^ Department of Pathology, Changhai Hospital, Navy Medical University, Shanghai, China; ^3^ Department of Urology, The Linyi People’s Hospital, Linyi, China

**Keywords:** neoleukin-2/15, OX40 ligand, cytotoxic activity, NK cell expansion, immunotherapy

## Abstract

Adoptive natural killer (NK) cell transfer has been demonstrated to be a promising immunotherapy approach against malignancies, but requires the administration of sufficient activated cells for treatment effectiveness. However, the paucity of clinical-grade to support the for large-scale cell expansion limits its feasibility. Here we developed a feeder-based NK cell expansion approach that utilizes OX40L armed NK-92 cell with secreting neoleukin-2/15 (Neo-2/15), a hyper-stable mimetic with a high affinity to IL-2Rβγ. The novel feeder cells (NK92-Neo2/15-OX40L) induced the expansion of NK cells with a 2180-fold expansion (median; 5 donors; range, 1767 to 2719) after 21 days of co-culture without added cytokines. These cells were highly cytotoxic against Raji cells and against several solid tumors *in vivo*. Mechanistically, NK92-Neo2/15-OX40L induced OX40 and OX40L expression on expanded NK cells and promoted the OX40-OX40L positive feedback loop, thus boosting NK cell function. Our data provided a novel NK cell expansion mechanism and insights into OX40-OX40L axis regulation of NK cell expansion.

## Introduction

As innate “sentinels”, NK cells serve an important role in the immune surveillance for pathogens and cancerous cells, implying that NK cells infusion is promising cellular immunotherapy for cancer (1). Therefore, the development of clinical-grade and low-cost approaches for large-scale NK cell expansion is essential to improve on its feasibility and clinical benefit. NK cells constitute approximately 5-25% of the lymphocytes with limited life-span, thus, obtaining enough cells is a major obstacle for NK cell immunotherapy, and the process is expensive and time-consuming.

Studies showed cytokines are essential in the induction of NK cell proliferation and cytotoxic activity (2–4). IL-2 or other cytokine combinations, such as IL-15 and IL-21, have been used to expand NK cell. However, natural cytokines only exhibit moderate affinity with IL-2Rβγ and are relatively unstable, thus the culture system requires continuous supplementation of cytokines, which greatly increases the cost of NK cell expansion. A recently designed IL-2 mimetics Neo-2/15 with a high affinity and specificity for IL-2Rβγ was reported to maintain T cell proliferation in kilo-picogram per milliliter scale (5). However, the effects of Neo-2/15 on NK cell expansion have not been reported.

Cytokine combination usually results in minimal NK cell expansion (1). One alternative approach would be to co-culture with feeder cells. K562 cells expressing costimulatory molecules in combinations with IL-2/15 greatly enhanced NK cell proliferation (6, 7). However, several studies reported that K562 reduced the cytotoxic activity of certain subpopulations of NK cells (8, 9). Therefore, it is necessary to find a more suitable feeder system for NK expansion. NK-92 is an NK cell line which considered to be a suitable allogeneic cell source for clinical application and its safety has been assessed in many clinical trials for tumor immunotherapy. The results imply that NK-92 can be suitable for NK cell feeders.

In this study, we found a novel approach to expand NK cells using NK-92 cells expressing OX40L and Neo-2/15, which enhanced *in vitro* and *in vivo* NK cell-mediated proliferation and cytotoxicity, respectively. Besides, we presented a possible mechanism of NK cell expansion through homotypic interaction of OX40-OX40L positive feedback loop.

## Materials and Methods

### Mice and Mice Models

NOD/SCID mice and nude mice were purchased from Cavens Experimental Animal Company (Changzhou, China). All mice were housed in specific pathogen-free conditions. Mice were maintained under a 12-hour light-dark cycle at 23°C, and had free access to water and standard rodent diet. Before surgery, mice were anesthetized with 2% isoflurane.

Lung cancer model: 50 μL HBSS containing 5×10^6^ A427 (luciferase Knock-in) cells were mixed with 50 μL Matrigel. The mixture was injected at a distance of 1.5 cm along the superior border of the rib into the left lung of male NOD/SCID mice, and unplugged after 10s.18 days after initial tumor growth, mice with similar tumor burden were selected by IVIS and randomly grouped.

Liver cancer model: HepG2 (luciferase Knock-in) cells were resuspended in HBSS with a density of 5×10^7^cell/mL. After anesthesia, male NOD/SCID mice were laterally laparotomized below the xiphoid to expose part of the liver, and 100 μL cell suspension was injected into the liver. Vetbond Tissue Adhesive (3M, 1469SB) was used to close the wound while withdrawing needle. The lobe was relocated followed by the closure of the peritoneum and abdominal wall. After 16 days of tumor growth, mice with similar tumor burden were randomly grouped.

Ovarian cancer model: CAOV3 (luciferase Knock-in) xenografts were obtained from subcutaneous tumor cells of female nude mice and prepared as 1mm^3^ blocks. A left subcostal incision was made to expose the left ovary of female NOD/SCID mice, and the xenograft was stitched to the ovarian capsule using 7/0 suture and sealed by Vetbond. After 25 days, mice with similar tumor burden were randomly grouped.

### Cells and Culture

Peripheral blood mononuclear cells (PBMCs) were isolated by Lymphoprep™ (StemCell, 07851) gradient centrifugation in SepMate™ (StemCell, 85450) Tubes. Primary Human NK cells were isolated from PBMCs by magnetic bead CD3 depletion (MiltenyiBiotec, 130-050-101) followed by CD56 (MiltenyiBiotec, 130-111-553) isolation. FACS analysis of anti-CD56 antibody revealed more than 90% purity of the NK cell population (Data not shown).

Purified NK cells were cultured in AIM-V Medium (GIBCO, 12055091) supplemented with indicated cytokines or feeder cells. 100ng/mL OKT3 (Muromonab) in the first culture cycle to deplete potential contaminated T or NKT cells.

NK-92 cells were purchased from American Type Culture Collection (ATCC). The complete medium for NK-92 was Alpha Minimum Essential medium (BasalMedia, L540KJ) with final a concentration of 0.2 mM inositol, 0.1 mM 2-mercaptoethanol; 0.02 mM folic acid, 20ng/ml recombinant IL-2 (Novoprotein, C013), 12.5% horse serum (Gibco, 26050088) and 12.5% fetal bovine serum (Gibco, 10091).

A427 was obtained from Boyu Biotechnology, while HepG2, CAOV3 and K562 were obtained from the Cell Bank of Type Culture Collection of the Chinese Academy of Sciences. A427, HepG2 and CAOV3 were cultured in α-MEM with 10% FBS. K562 was cultured in RPMI1640 with 10% FBS. A427, HepG2, and CAOV3 were labeled with luciferase (Genechem, GV633) as previously described (10).

### Generation of Engineered Cells Expressing Membrane-Bound Protein

OX40L (NM_003326.5) and 4-1BBL (NM_009404.3) sequences were fused with his-tag and then cloned into pLVX-EF1a-IRES-Puro (Addgene, 85132). The neo-2/15 sequence was fused with Flag-tag and cloned into pLVX-IRES-ZsGreen1 (Fenghui, BR021). The lentivirus was produced by co-transfection with the packaging plasmid psPAX2 and pMD2.G into lenti-X-293 cells cultured in DMEM medium with 10% FBS. The K562 or NK-92 were seeded into 24-well plates at 1×10^5^ cells/well and then infected at MOI=10. The cells were harvested 7 days post-infection and subsequently sorted by flow cytometry, resulting in GFP-positive or His-positive monoclonal cell lines. Anti-Flag-PE and anti-His-APC were obtained from BioLegend. NK92-based feeder cells were acclimatized in AIM-V medium after establishment.

### Cytotoxicity Assays

*In vitro* cytotoxicity assay of the expanded NK cells against target tumor cells (Raji, A427, HepG2 and CAOV3) was measured by FACS. Targeted cells were labeled using 0.5 μM CFSE (Sigma, 21888) at 37°C in PBS for 10min. 2×10^5^ CFSE-stained target cells were seeded into 96-well U-bottom plates in triplicate and subsequently mixed with expanded NK cells at the indicated effector-to-target ratios. Co-cultured cells were stained with 7-AAD (ThermoFisher, A1310) according to the manufacturer’s instructions. Dead target cells were detected as double-positive CFSE/7-AAD cells.

*In vivo* cytotoxicity assays were performed by quantification of xenograft tumor burden after NK cells administration. Optical images were analyzed using Living Image 4.3 software (PerkinElmer) as described previously (11).

### Western-Blot

The cells were lysed with cell lysis buffer (CST, 9803) supplemented with a protease inhibitor cocktail (Roche, 11873580001). The total protein concentration was measured using the BCA assay (Pierce, 23225). Proteins were separated by SDS-PAGE and then electrophoretically transferred onto nitrocellulose membranes. Subsequently, the blots were incubated with primary antibodies at 4°C overnight, then incubated with HRP conjugated secondary antibodies at room temperature for 2 hours. ECL reagent was used for imaging the blots (12). The antibody was obtained from CST: *#9936T* for NF-κB assay, *#41658* for TRAF5, *#15123* for OX40 and *#19541* for 4-1BB; #7074 and #7074 HRP-linked Abs for secondary antibodies.

### *Ex Vivo* Expansion of Human NK Cells

Purified NK cells were cultured in AIM-V Medium (GIBCO, 12055091) supplemented with indicated cytokines or irradiated feeder cells (1:5, 1000cGry for NK92 and 5000cGy for K562). Cultures were refreshed with half-volume media changes every three days and re-stimulated using feeder cells at a ratio of 1:1 every 7 days. When needed, a fraction of expanded NK cells were cryopreserved or discarded, and the remaining cells were carried forward for subsequent stimulations.

### Analysis of NK Cell Function

ELISA: The cell supernatant of feeder cells or cytotoxicity assay was collected to measure the levels of IFN-γ or Neo-2/15(Flag-tag) using their respective kits according to standard practice (R&D #DIF50C for IFN-γ, Abcam#ab125243 for Neo-2/15).

Flow cytometry: Flow cytometry was performed using the BD FACSAria system. Cells were incubated with indicated antibodies for 30 min at 4°C avoiding light. Cells were washed with DPBS and resuspended in FACS buffer before being subjected to FACS assay. Data were analyzed using FlowJo (version 10). The antibodies were from BioLegend and listed as follows: anti-CD107a-APC (328620), anti-OX40L-PE (326308), anti-CD56-PE (355504), anti-CD56-AlexaFluor488 (362518), anti-CD16-FITC (302006), anti-CD94-PE/Cy7 (305516), anti-NKG2A-APC (375108), anti-NKG2C-PE (375004), anti-NKG2D-FITC (320820), anti-KIR2DL2/L3-Percp/Cy5.5(312614), anti-KIR2DL1/S1/S3/S5-FITC (339504), anti-KIR3DL-BrilliantViolet421(312714), anti-NKp30-BrilliantViolet785 (325230), anti-NKp44-PE/Cy7 (325116), anti-NKp46-BrilliantViolet605 (137619).

### Regulatory Approvals

Peripheral blood used in this study was provided by M.G, C.S, Y.Q, L.Z and N.T in the author list (age 26~33). The experiments using primary NK cells were performed according to the guidelines of human subjects at Navy Medical University and approved by Scientific Investigation Board. All animal experiments were approved by the Scientific Investigation Board of Navy Medical University (Shanghai, China).

### Statistical Analysis

A two-tailed unpaired, student *t-test* was used to determine significance. One-way ANOVA with a Bonferroni post-test was used to compare the different groups. Survival analysis was performed by Kaplan-Meier survival analysis.

## Results

### The Stimulatory Effect of Feeder Cells With Secretory Neoleukin-2/15 on Human Natural Killer Cells

**Figure 1:**
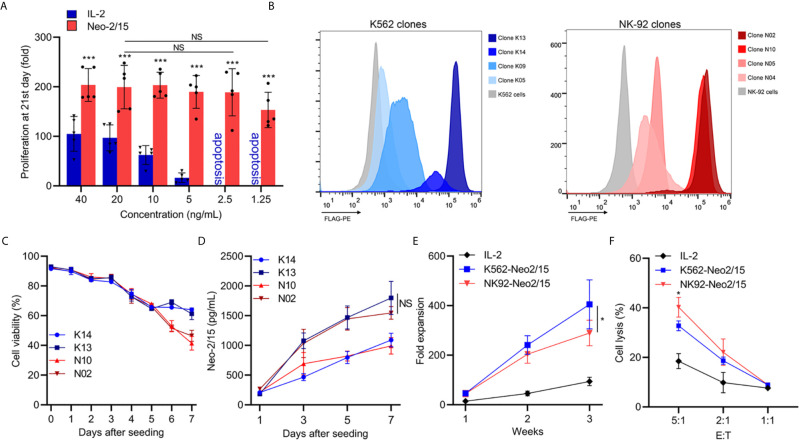
Generation and characterization of feeder cells expressing secretory Neoleukin-2/15 (*p<0.05, ***p<0.001; NS indicates no significant difference). **(A)** In vitro expansion of the NK cells from 5 volunteers by IL-2 or Neo-2/15 at the indicated concentration. **(B)** Genetically Engineered K562 or NK92 clones expressing secretory neoleukin-2/15 were analyzed by flow cytometry. **(C)** The survival of irradiated clones cultured in AIM-V medium and examined over one week. **(D)** Neo-2/15 secretion by indicated clones was measured. **(E)** NK cells were co-cultured with irradiated K562-Neo2/15 or NK92-Neo2/15, cell expansion fold was measured. **(F)** Cytotoxicity assay to measure NK-mediated cell killing in Raji cell at indicated effector-to-target ratios.

Neo-2/15 has been reported as a type of IL-2 mimic, which binds to human IL-2Rβγ with extremely high affinity (Kd=18.8nM) and cause significant expansion of T cells at low doses (5). To determine the Neo-2/15 effect on NK cell expansion, dose-dependent experiments were performed, and the fold expansion of NK cells with Neo-2/15 was higher than cells with IL-2, even at a concentration of 1.25ng/mL. This allowed NK cell expansion to solely rely on feeder-derived cytokines (**Figure 1A**).

To identify cell lines that could be beneficial in promoting NK cell expansion, K562 cells and NK-92 cells, stably transfected with Neo-2/15 lentivirus particles, were constructed and individual clones were picked. Neo-2/15 expression in the clones was identified by FACS. Clone NK92-N02 and N10, clone K562-K13 and K14 showed higher expression of Neo-2/15 (**Figures 1B, C** **)**. Then the life-span of irradiated feeder cells in the AIM-V medium was measured by CCK8 assay. The cell viability of irradiated N02 and N10 cells was slightly lower than that of K13 and K14 cells after 6 days (**Figure 1C**). However, Neo-2/15 production in the supernatants showed no significant difference (**Figure 1D**).

Subsequently, we compared the fold expansion and NK cells cytotoxicity after co-culture with either K562-Neo2/15 (K13) or NK92-Neo2/15 (N02) feeder cells. Compared with NK92-Neo2/15, the fold expansion of NK cells co-cultured with K562-Neo2/15 was slightly higher by the end of the third week (**Figure 1E**), but NK92-Neo-2/15 expanded NK cells had greater cytotoxicity (**Figure 1F**). A possible explanation is that K562 could produce minimal amounts of TGF-β after co-culture with NK-cells (**Supplementary Figure 1**).

### OX40L Armed Feeder Cells Improve NK Cell Expansion

**Figure 2:**
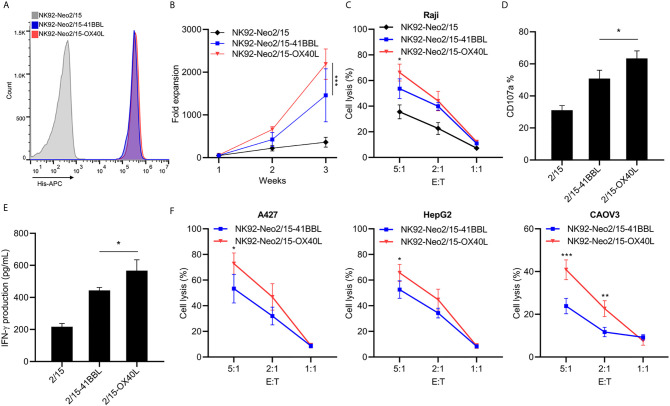
Generation and characterization of OX40L or 4-1BBL armed feeder cells (*p<0.05, **p<0.01, ***p<0.001). **(A)** Genetically knock-in OX40L or 4-1BBL in NK92-Neo2/15 clone N02 and analyzed by flow cytometry. **(B)** NK cells co-cultured with irradiated NK92-Neo2/15-41BBL or NK92-Neo2/15- OX40L, cell expansion fold was measured. **(C)** Cytotoxicity assay to measure NK92-Neo2/15-41BBL or NK92-Neo2/15- OX40L expanded NK cells in Raji cell. **(D)** NK cell activation of E:T=5:1 was determined by FACS staining for CD107a expression. **(E)** IFN-γ production in the supernatants of E:T=5:1 were evaluated by ELISA. **(F)** *In vitro* cytotoxicity assay to measure NK92-Neo2/15-41BBL or NK92-Neo2/15- OX40L expanded NK cells in A427, HepG2 and CAOV3 cells.

The optimal proliferation of NK cells requires both costimulatory signals and cytokines. TNFR superfamily mediated signal transduction has been reported to be involved in this process, including 4-1BB and OX40. To furtherly improve the expansion and cytotoxicity of NK cells, we transferred 4-1BBL or OX40L into NK92-Neo2/15 cells and selected two clones with similar expression levels (**Figure 2A**). The NK92-Neo2/15-OX40L-supported NK cells showed higher fold expansion compared to NK92-Neo2/15-41BBL-supported (1767-2719 *vs.* 833-2275 fold) at the third week (**Figure 2B**). Cytotoxicity of the expanded NK cells was then tested against Raji targets. Target cell lysis was significantly increased after NK92-Neo2/15-OX40L expanded NK treatment at a 5:1 E/T ratio (**Figure 2C**). Consistently, NK92-Neo2/15-OX40L-supported cells presented a higher positive rate of CD107a, and IFN-γ production in the supernatant (**Figures 2D, E** **)**. We evaluated the cytotoxicity of expanded NK cells from three donors against a panel of human tumor cell lines for liver cancer, lung cancer, and ovarian cancer cell lines. We found moderate to high cytotoxicity against all cell lines tested (**Figure 2F**).

**Figure 3:**
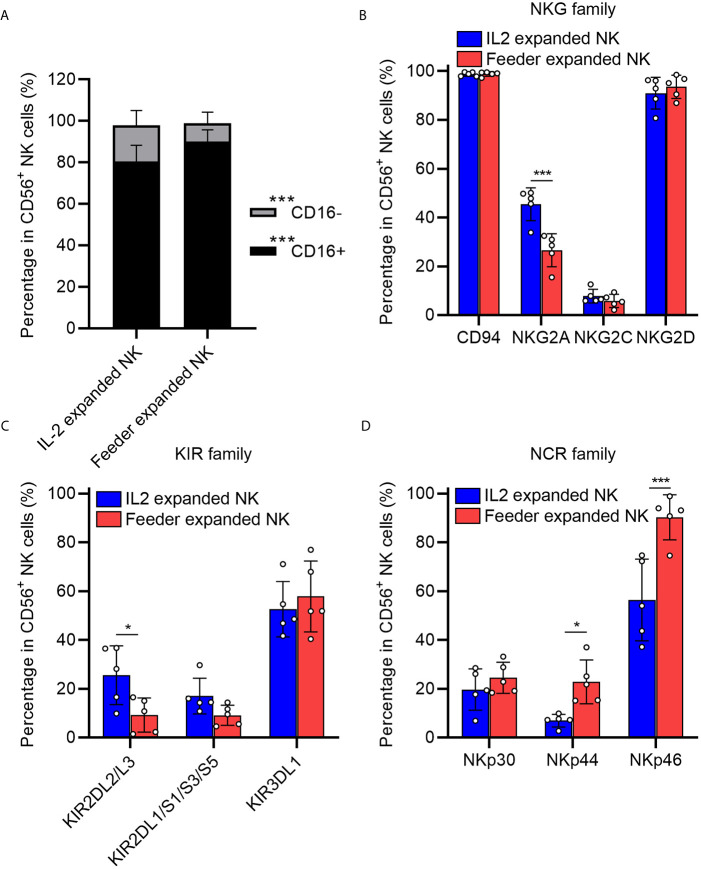
Phenotypic profiling of feeder expanded NK cells (*p < 0.05, ***p < 0.001). **(A)** Frequency of CD16^−^ and CD16^+^ NK cell subsets in the expanded cells. **(B)** Expression of natural killer receptors (NKG, CD94/NKG2A/NKG2C/NKG2D) in the expanded cells. **(C)** Expression of killer inhibitory receptors (KIRs, KIR2DL1/L2/L3/S1/S3/S5/KIR3DL1) in the expanded cells. **(D)** Expression of natural cytotoxicity triggering receptor (NCRs, NKp30/NKp44/NKp46) in the expanded cells.

We next examined the phenotypes of expanded NK cells expanded upon NK92-Neo2/15-OX40L or IL-2 treatment. The data indicated that proportions of CD16^+^ NK cells, which are responsible for cytotoxicity, increased in NK92-Neo2/15-OX40L group (**Figure 3A**). Two inhibitory receptors, NKG family member NKG2A and KIR family member KIR2DL2/L3, were less expressed in NK92-Neo2/15-OX40L group (**Figures 3B, C** **)**. NKp44 and NKp46 from NCR family which mediate cytolytic activity against target cells presented a higher expression in NK92-Neo2/15-OX40L group (**Figure 3D**).

### OX40 and OX40L Are Highly Induced on NK Cells During Expansion With NK92-Neo2/15-ox40l

**Figure 4:**
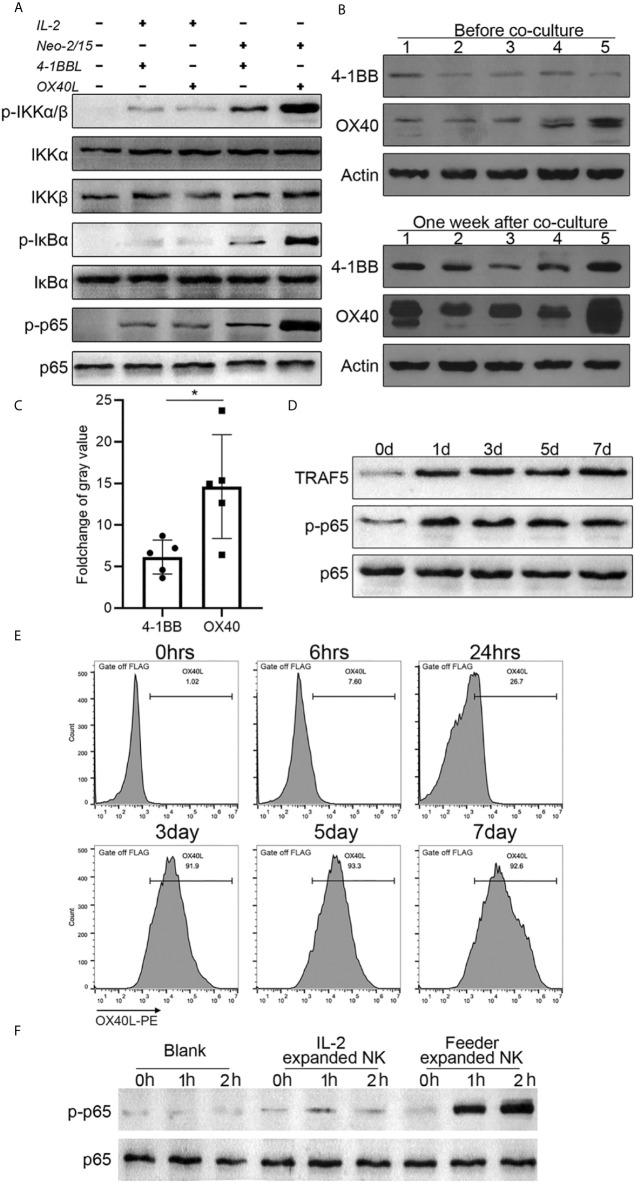
NK92-Neo2/15-OX40L promotes an OX40-OX40L positive feedback loop (*p < 0.05). **(A)** Western-blot detection of NK-κB activation for indicated treatment. **(B)** 4-1BB and OX40 expression in expanded NK cells from 5 donors, measured by Western-blot before and after feeder cell treatment. **(C)** Relative gray value statistics of protein level in **(B)**. **(D)** Immunoblot of TRAF5, phosphorylated-p65, and p65 was detected. **(E)** FACS analysis of OX40L expression on expanded NK. **(F)** NK cells from PBMC, treated by IL-2 or NK92-Neo2/15-OX40L expanded NK cells at a ratio of 19:1, freshly collected NK cells were used as control. Phosphorylated p65 were determined by western blot.

To gain insights into the mechanism of NK cell proliferation mediated by OX40L, 4-1BBL or OX40L were overexpressed in NK-92 cells, and two clones with similar expression levels were selected (**Supplementary Figure 2A**) and the doubling time of feeder cells was shown in **Supplementary Figure 2B**. The cells were used as feeders and combined with IL-2 or Neo-2/15 to treat NK cells. Western blotting analysis showed that neither OX40L nor 4-1BBL modified feeder was able to effectively activate NF-κB when combined with IL-2. Notably, NK92-OX40L can more strongly activated NF-κB when combined with Neo-2/15 (**Figure 4A**). Subsequently, Neo-2/15 stimulated NK cells from five donors, then 4-1BB and OX40 expression was determined by western blot analysis. Neo-2/15 was found to strongly induce the OX40 expression on the NK cell surface (**Figure 4B**). Although 4-1BB expression was also promoted, the fold-change was significantly lower compared with OX40 (**Figure 4C**). In addition, immunoblotting was performed to detect TRAF5, p-p65, and total p65 in OX40 cascade at indicated day after NK92-Neo2/15-OX40L feeder co-culture. Continuous activation of OX40 cascade in expanded NK cells is shown in **Figure 4D**.

To dissect the mechanism of OX40/OX40L interaction, flow cytometry was performed to investigate the OX40L expression following NK92-Neo2/15-OX40L co-culture. After three days of co-culture, more than 90% of expanded NK cells had positive OX40L expression (**Figure 4E**). Finally, we added IL-2 expanded cells or NK92-Neo2/15-OX40L expanded cells to Neo-2/15 pre-treated NK cells. Immunoblotting showed that NK92-Neo2/15-OX40L-supported cells significantly promoted p65 phosphorylation (**Figure 4F**), further demonstrating that NK92-Neo2/15-OX40L induced OX40 and OX40L expression on NK cells and promoted OX40-OX40L positive feedback loop. Importantly, we compared NK92-Neo2/15-OX40L expansion ability with NK92-OX40L plus IL-2/IL-15 or NK92-OX40L plus IL-2/IL-21, results indicated NK92-Neo2/15-OX40L performed maximum expansion (**Supplementary Figure 3**).

### NK92-Neo2/15-OX40L Improves NK Cells Antitumor Activity *In Vivo*

**Figure 5:**
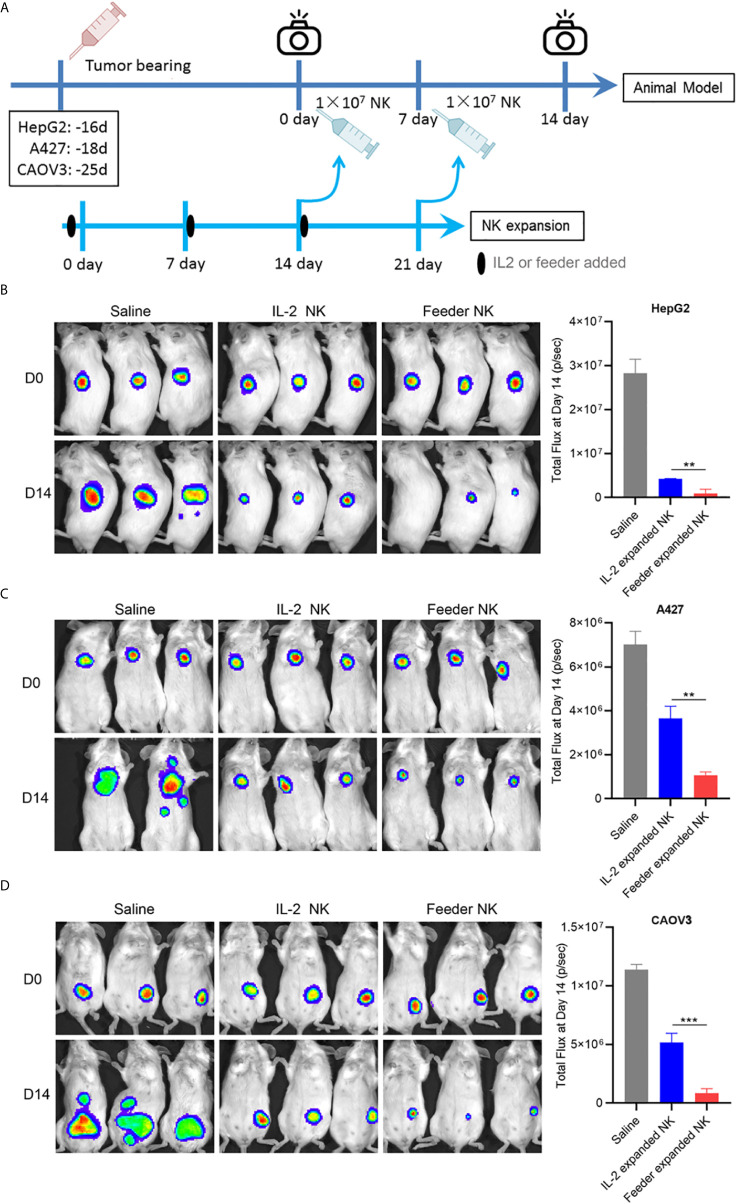
NK92-Neo2/15-OX40L improves antitumor activity of NK cells *in vivo* (**p < 0.01, ***p < 0.001). **(A)** Experimental setup schematics. **(B)** NOD/SCID mice treated as in **(A)**, HepG2 tumors were visualized by IVIS SpectrumCT, and the tumor size was assessed by bioluminescence. **(C)** NOD/SCID mice treated as in **(A)**, A427 tumors were visualized by IVIS SpectrumCT, and the tumor size was assessed by bioluminescence. **(D)** NOD/SCID mice treated as in **(A)**, CAOV3 tumors were visualized by IVIS SpectrumCT, and the tumor size was assessed by bioluminescence.

To evaluate the therapeutic efficacy of NK92-Neo2/15-OX40L expanded NK cells *in vivo*, killing assays were performed on HepG2 (liver cancer), A427 (lung cancer) and CAVO3 (ovarian cancer) derived xenograft models. Here, we compared IL-2 expanded cells and NK92-Neo2/15-OX40L expanded cells. To allow the most consistent comparisons between multiple cancers and across multiple tumors bearing mice, NK cells from the same donor were used. The expansion began 14 days before modeling, and tumor growth was monitored by bioluminescent imaging (**Figure 5A**). Compared with untreated tumor-bearing mice, three murine models treated with NK cells showed a significant reduction in tumor burden after 14 days, with maximal anti-tumor activity seen in NK92-Neo2/15-OX40L-supported cells (**Figures 5B–D**). Increased numbers of infiltrating CD56^+^ cells were also present in the Feeder-expanded NK treated xenografts (**Supplementary Figure 4**).

## Discussion

Numerous studies of adoptive transfer of NK cells have indicated responses in a wide range of malignancies (13, 14). However, the large-scale clinical application of NK cell immunotherapy has been limited by the lack of robust and low-cost methods for cell expansion. Here, we designed and evaluated a novel cytokine-free method using feeder cells to expand human PBMC-derived NK cell’s proliferation ability and cytotoxic function.

IL-2 family cytokines are essential for the proliferation and activation of NK cells (2–4). IL-2 is the most commonly used cytokines for NK cell expansion, but IL-2 dose potentially promotes Treg expansion, which contributes to tumor tolerance (4). Substitution of cytokines such as IL-15 or IL-21 may also help in Treg expansion, but inducible NKG2A expression can weaken NK cell cytotoxicity (15). In addition, natural IL-2 family cytokines lack both stability and high affinity to IL-2Rβγ, resulting in continuous supplementation of cytokines in long-term cultivation. Neo-2/15 is reported to be a hyper-stable artificial peptide, binding human IL-2Rβγ with higher affinity than the natural cytokines, and maintains T cell proliferation at a concentration of 3.1 ng/mL (5). Our results showed that Neo-2/15 can significantly promote NK cell proliferation even at a concentration of 1.25ng/mL, while a similar concentration of IL-2 could not even maintain NK cell survival. This data allowed us to consider the use of feeder cells to maintain a cytokine-free culture system.

Several cancer cell lines, like human myelogenous leukemia K562 and human Burkitt’s lymphoma cell line Daudi, have been used as feeder cells for NK expansion (6, 7). Nevertheless NK-92, an NK cell line assessed in numerous clinical immunotherapy trials, has not been used as feeder cells. In this study, we used NK-92 as a candidate for expanding NK cells and compared it with K562. Although the NK92-based feeder showed slightly lower expansion potential than K562, the expanded cells had higher cytotoxic activity. This difference may be related to the expression of TGF-β in K562 cells, which is consistent with a previous study reporting that K562 reduced the cytotoxic activity of certain subpopulations of NK cells (8, 9). Therefore, although NK-92 as a feeder cell has a slightly lower ability to expand NK cells than that K562, it has significant advantages in enhancing cytotoxic activity.

IL-2R cascade appears to be necessary but not the only source for NK expansion, and costimulatory signals are also required for optimal proliferation (16). TNFR superfamily ligands (TNFRSFL) are key co-stimulants for the preferential expansion of NK cells. Fujisaki H et al. reported a 21.6-fold expansion of NK cells after co-culture with modified K562 to express membrane-bound form IL-15 and 41BBL (7). In addition, OX40L, a member of TNFRSF, has also been reported to promote NK cell function *via* the OX40 cascade (17–19). Based on NK92-Neo2/15, we furtherly constructed two feeder cells NK92-Neo2/15-41BBL and NK92-Neo2/15-OX40L. Results showed that NK92-Neo2/15-OX40L expanded NK cells not only exhibited higher proliferative and cytotoxic capacity but also mediated significantly higher secretion levels of IFN-γ when compared to NK92-Neo2/15-41BBL expanded cells. OX40L and Neo2/15 were found to be the best combination for NK cell production and expansion.

Studies have shown that both 4-1BB and OX40 are expressed on the surface of activated NK cells, and their expression intensity is regulated by activation signals (17, 20). Our study found that the expression of OX40 in NK cells was strongly induced by Neo-2/15 compared with the 4-1BB signal. This phenomenon can partially explain why is OX40L is more efficient in promoting NK cell proliferation. However, feeder cells added weekly may not provide sufficient OX40L for rapid cell proliferation. Previous studies reported inducible OX40L expression on activated NK cells (21). Consistently, in this study, we observed the same phenomenon that NK92-Neo2/15-OX40L induced the expression of OX40L on NK cells. Over 90% of NK cells were OX40L positive after co-culture. Notably, co-incubation of PBMC-derived NK cells with feeder-expanded NK could also activate the NK-κB pathway. This evidence indicates that NK92-Neo2/15-OX40L significantly induces the expression of OX40 and OX40L on the surface of NK cells and form a positive feedback loop.

Solid tumors, unlike hematological malignancies, have a complex immune microenvironment which blocks NK cells infiltration and subsequently caused the limited efficacy against solid tumors (13). Our *in vivo* results showed that NK92-Neo2/15-OX40L expanded NK cells showed stronger infiltration ability and initiate a marked higher antitumor effect compared with IL-2 expanded NK cells. In conclusion, the NK92-Neo2/15-OX40L feeder triggers the expansion of NK cells with highly cytotoxicity through the formation of an OX40L/OX40 positive feedback loop. Therefore, this study provides a novel NK cell expansion mechanism and insights into OX40-OX40L axis regulation of NK cell expansion.

## Data Availability Statement

The raw data supporting the conclusions of this article will be made available by the authors, without undue reservation.

## Ethics Statement

The studies involving human participants were reviewed and approved by Scientific Investigation Board of Navy Medical University. The patients/participants provided their written informed consent to participate in this study. The animal study was reviewed and approved by Scientific Investigation Board of Navy Medical University.

## Author Contributions

YL, FG, and JZ designed this study and supervised the research. MG and CS performed the research, analyzed data, and wrote the paper. YQ contributed in animal model and IVIS. LZ and NT contributed in technical support. GW contributed in improvement of language and artwork. All authors contributed to the article and approved the submitted version.

## Funding

This work was supported by the National Natural Science Foundation of China (No.82071799, No.81972683, and No.81971503) and Shanghai Science and Technology Development Funds (No.20ZR1469900).

## Conflict of Interest

The authors declare that the research was conducted in the absence of any commercial or financial relationships that could be construed as a potential conflict of interest.
