# Supplementary material for: Proliferation of Highly Cytotoxic Human Natural Killer Cells by OX40L Armed NK-92 With Secretory Neoleukin-2/15 for Cancer Immunotherapy
Source: Front Oncol. 2021 Apr 15;11:632540. doi: 10.3389/fonc.2021.632540 (PMC8083131; doi:10.3389/fonc.2021.632540)
Supplement: Supplementary file 1 [file DataSheet_1.docx]

Supplementary Material





**Supplementary Figure 1.** Expression of TGF-β in K562 cells were measured by flow cytometry.


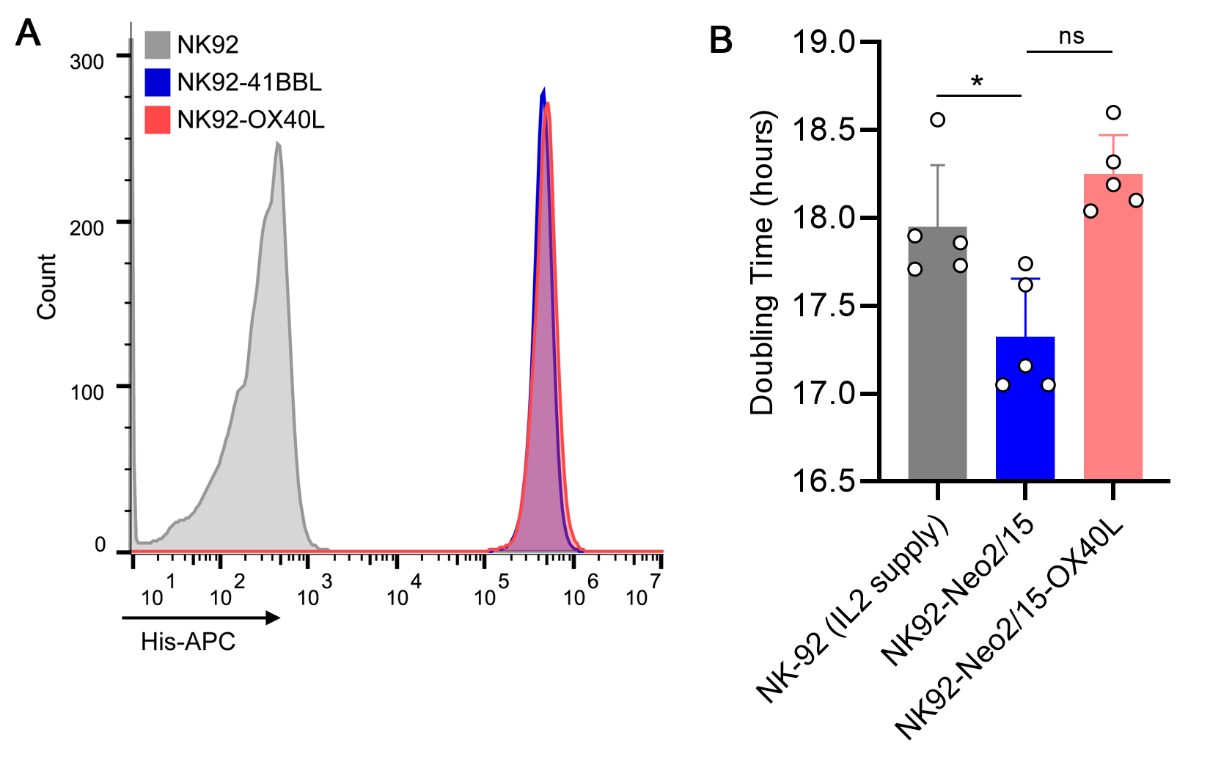


**Supplementary Figure 2.** (A) Expression of 41BBL or OX40L in NK-92 cell clones was identified *via* flow cytometry by labeling His-tag. (B) Cell doubling times were calculated using online population doubling time software (http://www.doubling-time.com/compute_more.php). *p< 0.05; ns, Not significant.


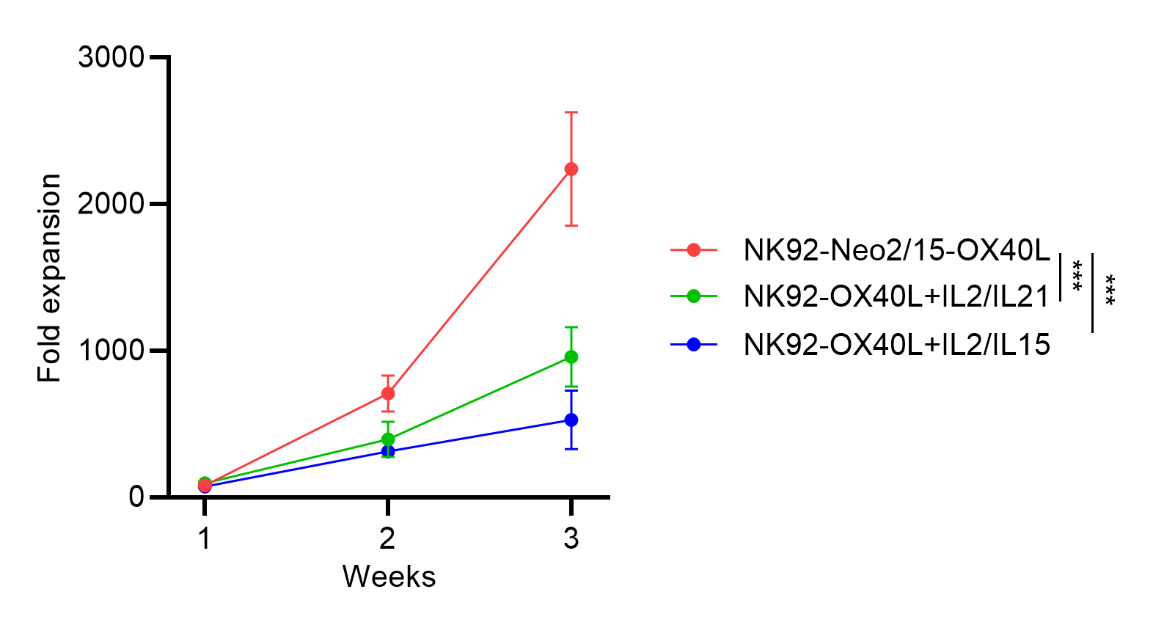


**Supplementary Figure 3.** NK cells from five donors co-cultured with irradiated NK92-Neo2/15-OX40L or NK92-OX40L plus IL2/IL15(20+20ng/mL) or NK92-OX40L plus IL2/IL15(20+20ng/mL), cell expansion fold was measured. *** p< 0.001.


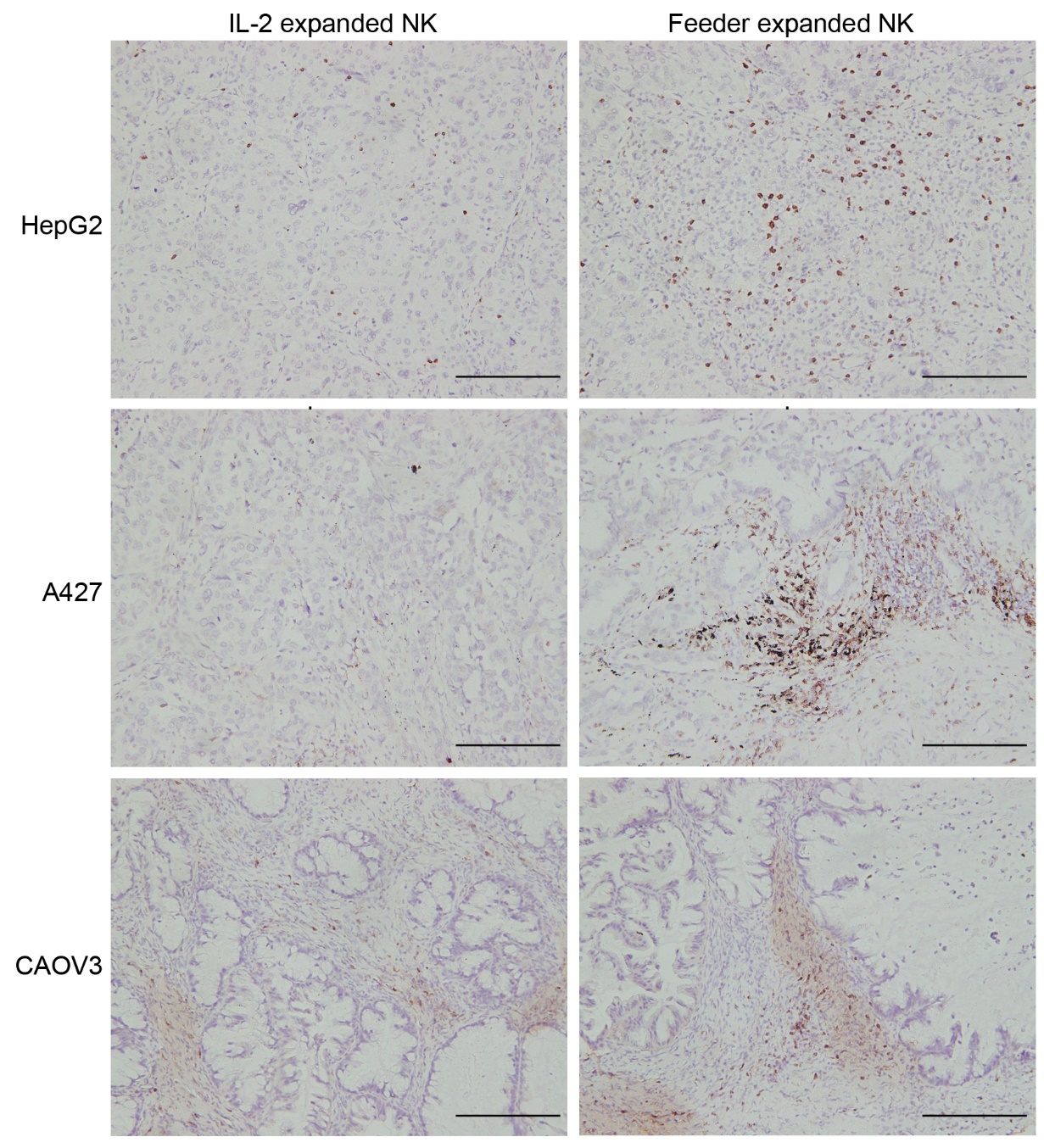


**Supplementary Figure 4.** Human CD56 immunohistochemical (IHC) stain for assessment of natural killer cells infiltration in HepG2, A427 or CAOV3 xenografts after NK administration (×200, scale bar=200 μm).
